# Environmental and socio-economic determinants of fecal sludge emptying in Sub-Saharan Africa: A cross-sectional mixed-methods study in Abidjan, Côte d’Ivoire

**DOI:** 10.1007/s11356-024-35631-6

**Published:** 2024-12-05

**Authors:** Lou Tinan Ange-Laetitia Tra, Kouassi Dongo, Vitor Pessoa Colombo, Shirish Singh, Jérôme Chenal

**Affiliations:** 1 Département Recherche et Développement (DRD), Centre Suisse de Recherches Scientifiques en Côte d’Ivoire (CSRS), 01 BP 1303 Abidjan 01, Côte d’Ivoire; 2 Laboratoire des Sciences du Sol, de l’Eau et des Géo Matériaux (LSSEG), Ecole Doctorale STAD, Université Félix Houphouët-Boigny, 01 BP V34 Abidjan 01, Côte d’Ivoire; 3 Communauté d’Etudes pour l’Aménagement du Territoire, Ecole Polytechnique Fédérale de Lausanne (EPFL), Bâtiment BP - Station 16, CH-1015 Lausanne, Switzerland; 4 IHE Delft Institute for Water Education, PO Box 3015, 2601 DA Delft, The Netherlands; 5 Center of Urban Systems (CUS), University Mohammed VI Polytechnic (UM6P), 43150 Benguerir, Morocco

**Keywords:** Non-sewered sanitation, Fecal sludge emptying, Environmental pollution, Urban health, WASH, Côte d’Ivoire

## Abstract

This study sought to understand the determinants of household’s choices for fecal sludge (FS) emptying to help fighting against unhygienic/unsafe practices and environmental pollution. A cross-sectional mixed study used in the municipality of Yopougon (Abidjan, Côte d’Ivoire), involved surveys (559 household), and 24 semi-structured interviews. Adjusted odds ratios (*aORs*) from logistic regressions indicated the variables significantly associated with these practices while thematic content analysis helped assessing stakeholders’ perceptions. Respondents (47.6%) reported unhygienic/unsafe emptying, mainly direct FS discharge into open drains or the immediate environment via illegal toilets/pits connections (38.1% out of 47.6%). Household heads with a high monthly income (*aOR* = 0.59; 95% *CI* 0.35–0.98; *p* = 0.04), house ownership (*aOR* = 0.27; 95% *CI* 0.15–0.47; *p* < 0.001) and distance to a water body (*aOR* = 0.03; 95% *CI* 0.009–0.12; *p* < 0.001) were the main explanatory variables, significantly reducing the likelihood of unsafe emptying. This likelihood increases when the household is close (≤ 100 m) to gully/gutter (*aOR* = 1.73; 95% *CI* 0.99–3.03; *p* = 0.05). Our recommendations for improving sanitation in low-income settings include reducing environmental pollution caused by unhygienic/unsafe emptying practices requires, enhancing and enforcing current regulations and making implementing texts effective, raising public awareness focusing on the benefits associated to hygienic sanitation practices, and subsidizing sanitation interventions for the poor that includes homeowners.

**Supplementary Information:**

The online version contains supplementary material available at 10.1007/s11356-024-35631-6.

## Introduction

About 3 billion people worldwide, or 46% of the world’s population, do not have access to safely managed sanitation services (WHO/UNICEF 2021). This situation is more alarming in Sub-Saharan Africa, where over 60% people have no access to basic sanitation services, and nearly 70% are served with non-sewered sanitation systems (WHO/UNICEF 2021). According to Strande and Brdjanovic (2014), these systems produce fecal sludge (a mixture of excreta and black water, with, or without gray water) that still require proper treatment before being disposed. This sludge is not transported through a sewer system but rather stored on site, where it can undergo primary treatment. Various types of on-site containment are used to store septic sludge. They include septic tanks and pit latrines that periodically require the emptying of fecal sludge (when full), which need to be transported to treatment stations that preserve healthy environmental conditions. Emptying consists of the collection of the fecal matter from the storage container, eventually including transport to a treatment facility.

The unsanitary disposal of fecal sludge is a matter of serious concern (Singh et al. 2020). These emptying services remain a major challenge for low- and middle-income urban households, particularly in informal settlements (Lerebours et al. 2022; Thye et al. 2009).

In Sub-Saharan Africa, the most commonly recognized emptying options are mechanical and manual systems (Muoghalu et al. 2023). Mechanical emptying employs sophisticated means such as emptying trucks and hydro-cleaners to extract and transport fecal sludge to treatment plants (Seleman et al. 2020). Meanwhile, manual emptying is generally practiced with rudimentary tools, often without adequate personal protection, exposing emptier to enormous health risks (Chowdhry and Kone 2012). Moreover, when manually emptied, fecal sludge is often disposed of in the surrounding environment without any treatment, leading to pollution of soil and water resources (Peletz et al. 2020). Hygienic/safe emptying could be defined as a practice where fecal sludge is collected, transported, and disposed of in appropriate places (with proper treatment), whereas unhygienic/unsafe emptying practice involves direct contact with fecal matter during emptying and/or discharge of fecal sludge into the environment without any treatment. Unhygienic/unsafe emptying is often conducted manually and is generally prohibited by local governments (Strande and Brdjanovic 2014).

In addition to mechanical and manual emptying methods, fecal sludge can also be removed from the tanks/pits through deliberate practices involving illegal connections of tanks/pits to a receiving environment, e.g., a river or open green area (Ouattara et al. 2021). These practices, which could be perceived as a means of evacuating only the effluent or the overflow from the pits, are increasingly used as methods of gradually evacuating all the fecal matter produced (effluent and sludge). These methods although passive, are comparable to emptying practices, and precisely unhygienic/unsafe fecal sludge emptying methods. In some cases, households do not have pits where the sludge can accumulate, before being discharged, so they discharge it directly into the nature through improvised pipes. The latter usually connect the pits to nearby gullies, channels, or other water bodies. Despite legal measures to ensure hygienic/safe practices, unsafe methods to empty fecal sludge are still used by many households in low- and middle-income countries (Conaway et al. 2023).

The specific reasons why households engage in unhygienic/unsafe practices have not been sufficiently studied, notably whether there are environmental conditions that may motivate such unsafe methods. Filling these gaps with empirical evidence would be an important step towards informing decision-makers on ways of improving emptying practices for proper management of non-sewered sanitation. According to a recent review (Muoghalu et al. 2023), accessibility by trucks and costs of emptying have been identified as the most important factors influencing households in developing countries to engage in hazardous emptying practices. More empirical studies are required to better understand the drivers of these hazardous emptying practices, including the environmental and socio-economic characteristics of the settings in which they are more likely to happen.

In Côte d’Ivoire, access to sanitation remains a critical issue for public health and sustainable development. Despite numerous efforts to improve sanitation in recent decades, in 2022, only 37% of the population had access to safely managed sanitation (WHO/UNICEF 2022). In fast-growing metropolitan and urban areas, a large proportion of the population, especially in informal settlements, struggles to access adequate sanitation (Angoua et al. 2018). Rapid urbanization, risen from 32% in 1975 to 52.5% in 2021 (INS 2022), has indeed aggravated the situation, with increasing pressure on existing infrastructures. While previous studies have emphasized the lack of sanitation services in precarious, so-called “informal” settlements (Cherunya et al. 2020; Pessoa Colombo et al. 2023a, b) one may also find significant challenges in “formal” areas that do not respect existing regulations, leading to equally significant environmental health hazards.

On this basis, we conducted a cross-sectional mixed-methods study to assess, in the context of a large Ivorian city: (i) the occurrence of unhygienic/unsafe emptying practices, (ii) environmental and socio-economic factors associated with these practices, and (iii) household’s characteristics associated with unhygienic/unsafe emptying practices.

## Materials and methods

### Geographic scope

Our study site is located in Abidjan, the economic capital of Côte d’Ivoire. With its many assets (port, airport, factories, etc.), this city has positioned itself as a major economic center where huge subregional and even international trade transactions take place (Dongo et al. 2009). Abidjan has attracted many internal and external migrants, resulting in rapid demographic growth. This has led to the simultaneous development of both formal and unplanned neighborhoods, resulting in a diverse urban fabric accompanied and a variety of sanitation solutions (not always safe). With a sewerage coverage rate of 40%, the majority of Abidjan’s population (60%) relies on unsewered systems to dispose of wastewater and excreta (USAID 2020).

The study area is part of a coastal sedimentary basin. The lithological environment is dominated by alternating sediments of clays, clayey sands and more or less ferruginous sandstones. The permeability of these layers varies from 10 − ^3^ m/s to 10 − ^6^ m/s, depending on whether we are in coarse fine sands or in the presence of a facies variation (Aghui and Biémi 1984; Jourda 1987). In the study area, the piezometric level of the water table is on average of 3.6 m below the ground surface (Ahoulé et al. 2017). These conditions lend themselves to the installation of on-site sanitation systems for the removal of wastewater and excreta.

**Fig. 1:**
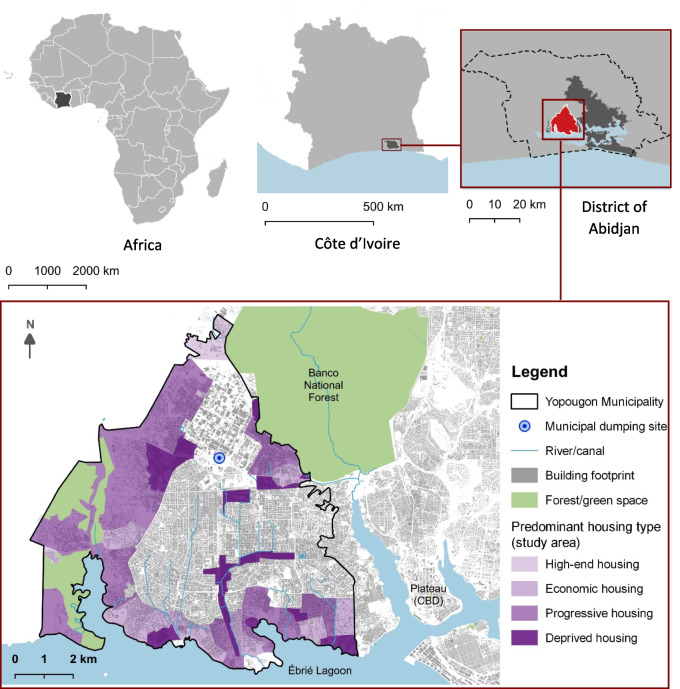
Map of the municipality of Yopougon (Abidjan, Côte d’Ivoire) showing the study area subdivided into zones based on the predominant housing type

We conducted our fieldwork in the municipality (“Commune”) of Yopougon, which is one of ten municipalities of Abidjan, being located in the western part of Abidjan, bordering the Ebrié lagoon (Fig. 1). It is the largest and most populated municipality in Abidjan, with an estimated population of 1,571,065 inhabitants (INS 2022) and a land coverage of approximately 153.06 km^2^. In Yopougon, although 60% of the households are served by a sewer network located in the central part of the municipality, about 40% are still using non-sewered sanitation (BRL 2021). Households served with non-sewered sanitation often face issues to empty fecal sludge from their containment structure — pit latrines or septic tanks — when full. In the study area, three types of on-site sanitation systems (OSS) are generally found. The most dominant are lined pits similar to septic tanks, characterized by two compartments (3/4 and 1/4), as described by Tilley et al. (2014). The second most common structures are septic tanks connected to an infiltration well. In third place are unlined pits, including latrine pits.

These OSS are emptied mechanically through tanker trucks, or manually by well-diggers (S. L. Coulibaly et al. 2016). Alongside these two practices, many households are increasingly resorting to other methods to empty fecal sludge. Some households connect their pits directly to nearby open drains, while others use motor pumps to empty their pits. Of all these practices, the use of a tanker truck is the only one considered hygienic/safe at national level. Such mechanical method avoids contact with fecal sludge, which is transported to a designated dumping site managed by the central sanitation authorities — the case of Yopougon — or to an appropriate treatment station (Thye et al. 2011). In contrast, manual emptying or emptying using a motor pump, as well as direct discharge of fecal sludge into open drains or the immediate environment via illegal toilet/pit connections are considered unhygienic/unsafe (Capone et al. 2020).

The outskirts of central Yopougon compose the area served by non-sewered sanitation and divided into four zones based on the predominant housing type (Fig. 1). These four housing types, (i) high-end housing; (ii) economic housing; (iii) progressive housing, and (iv) deprived housing, have been categorized according to UN-habitat’s description (ONU-Habitat 2012), as summarized in Table 1.

**Table 1:** Description of housing types in Yopougon according to UN-habitat (ONU-Habitat 2012)

Types of housing	Description
Deprived housing	- Generally formed around courtyards with shared toilets, accommodating several households, and often made of rudimentary materials (plastic, wood, etc.). This type of housing is characterized by overcrowding and spatial congestion, making it difficult to access. They lack basic sanitation services
Progressive housing	- Also organized around courtyards and often with shared toilets, but sometimes with single-family compounds. It is characterized unfinished structures (progressive building) but that employ proper construction materials. Its main difference with deprived housing is the presence of basic sanitation infrastructures and roads accessible by cars (but in poor condition)
Economic housing	- Apartments or detached houses where toilets are not shared and reachable through roads accessible by cars (sometimes paved, but in poor condition)
High-end housing	- Comfortable, single-family villas and apartments with non-shared toilets. Main and secondary roads are paved and in good condition

### Data

This research combined primary and secondary datasets. The former consisted of household surveys and interviews, respectively collected using questionnaires through the KoboCollect mobile application (version 2022.4.4), and semi-structured interviews addressed to key informants in the sanitation sector. As for the secondary data, it consisted of the building footprints map of Yopougon from 2015 obtained from the *Bureau National d’Études et Travaux* (BNETD). This dataset was used to generate quantitative indicators describing the form of the built environment in the study area. The 2015 map was manually corrected and updated using QGIS 3.24.2 software, based on very high-resolution satellite images (± 50 cm/pixel).

#### Household surveys

Household data collection took place from October to December 2022. A pilot survey was conducted to test the validity and applicability of the questionnaire. Its final version was structured into two sections: the first included basic information on socio-economic and environmental characteristics; the second focused on sanitation and hygiene services. The list of variables observed in the household surveys is given in the quantitative analysis section. The sample size was obtained by applying Eq. (1) (Rea and Parker 2014):

1 $${\varvec{n}}=\frac{{{\varvec{t}}}^{2}{\varvec{P}}\left(1-{\varvec{P}}\right){\varvec{x}}{\varvec{N}}}{({{\varvec{t}}}^{2}{\varvec{P}}\left(1-{\varvec{P}}\right)+\left({\varvec{N}}-1\right){\varvec{x}}{{\varvec{Y}}}^{2})}$$

where*** n*** is the minimum sample size required; ***N*** is the size of the target population (in this case, counted in number of households, here *N* = 349 480 households); ***t*** is the desired confidence level (for a confidence level of 95% as considered in this study, *t* = 1.96); ***Y*** is the tolerated margin of error corresponding to the statistical risk (here, 5%); ***P*** is the expected proportion of the outcome of interest in the target population (*P* = 50% if no previous information is available, which was the case in this study). Applying Eq. (1) with a 5% margin of error and 95% precision resulted in a minimum size of 384 households to cover the entire study area. A percentage of 80% of respondents was considered sufficient for each interviewer, hence:

2 $${\varvec{n}}\boldsymbol{^{\prime}}=\frac{{\varvec{n}}}{{\varvec{r}}}$$

where “*r*” is the expected percentage of respondents; “*n*” is the size of the population to be surveyed and, “*n*” is the minimum size of the population to be interviewed. Thus, considering the percentage of non-response (or “wastage”), the total number of households to be interviewed should be 480. However, to strengthen the representativeness of the sample (quality control), and based on the resources available, additional households (*n* = 79 households) were interviewed, and the final sample size was 559 households. To consider the different social strata, this sample was subsequently divided proportionally into 4 homogeneous subgroups corresponding to the 4 types of housing (described in Table 1) that characterized the population of the study area. In each subgroup, we selected households through a simple random sampling.

The household survey was administered by six enumerators who were trained to be familiarized with the questionnaire and the mobile application used. In Sub-Saharan Africa, women are usually responsible for sanitation management at the household level (Assefa et al. 2021). Therefore, the household surveys targeted females, but responses were primarily requested from the person effectively responsible for paying for the emptying service. When this person was absent, the head of the household or any other representative of the household (older than 18 years) could answer the questions. For each survey, the geolocation of the household was recorded. Each enumerator was equipped with a tablet (Samsung Galaxy Tab A/LTE tablet) that enabled him/her to administer the questionnaire and geolocate the household via the Kobo Collect application (using the GPS integrated in the tablet).

#### Characterization of the built environment

One of the main knowledge gaps addressed by this study was to assess whether unhygienic emptying practices are associated with environmental (spatial) conditions. Therefore, the survey included density and spatial organization of buildings as key exposure variables — hereafter denominated as “morphological indicators.” As performed in a previous study on environmental determinants of access to sanitation (Pessoa Colombo et al. 2023a, b), these morphological indicators were defined as quantitative variables derived from building footprints (see Table 2). They were calculated in Python language, using the Momepy package (Fleischmann 2019). Two geographic scales of analysis, namely, smaller (building level) and larger (block level), were considered in the definition of these indicators. In this study, given that the building footprint map was updated manually, the spatial analysis did not consider the mean deviation between neighboring structures — as, indeed, the manual digitization could result in inaccurate building orientations, eventually biasing the results towards a higher entropy than the real situation on site. Hence, the morphological indicators focused on levels of land occupation, size, and number of buildings.

**Table 2:** List of morphological indicators, by geographic scale (object and block level)

Variable	Level	Description/calculation
Voronoi cell	Object (building)	Voronoi tessellation cell obtained from the buildings’ footprints, generating a plot-like structure
Covered area ratio	Object (building)	$$\frac{Area of building footprint}{Area of tessellation cell}$$
Zonal, mean covered area ratio	Block (500 m radius)	Iterative calculation: for each building, get the mean covered area ratio of all neighbors within 500 m
Number of neighboring structures	Block (500 m radius)	Iterative calculation: for each building, count the number of structures (building footprints within 500 m

#### Semi-structured interviews

We conducted a series of semi-structured interviews (*n* = 24) with various stakeholders between 16 and 22 May 2023. We used the purposive sampling method (Gill 2020) to select the interviewees. This approach involves selecting the most relevant participants for the study based on the purpose of the research being conducted and the information to be collected. In this study, interviewees were selected based on their level of interest and/or influence in fecal sludge management, with a particular focus related to pit emptying practices in the study area. Based on these criteria, institutional, technical, and community stakeholders were selected: (i) resource persons from public sanitation institutions, including the National Office of Sanitation and drainage (*Office National de l’Assainissement et du Drainage*: ONAD) and the Municipality of Yopougon, (ii) emptying service providers (truck owners and drivers), and (iii) service users (community leaders or union officials in the respective housing types). In each of these, 3 stakeholder groups, at least 2 resource persons were enrolled to obtain the maximum amount of information and verify its accuracy (see Table 3).

**Table 3:** List of stakeholders interviewed

Structure	Person/function		Number
ONAD	Sanitation officer in charge of non-sewerage sanitation		1
	Sanitation officer in charge of monitoring and evaluation		1
Yopougon Municipality	Technical director of the municipality		1
	Officer in charge of WASH		1
Services providers	Truck owners		5
	Truck drivers		7
Community leaders	Sub-district chief	Living in high-end housing	2
		Living in economic housing	2
		Living in progressive class housing	2
		Living in deprived housing	2

The interviews focused on (1) actors involved in fecal sludge management (FSM), their role and level of collaboration; (2) main challenges related to FSM; (3) different practices to empty pit latrines and septic tanks; (4) level of knowledge on health and environmental risks related to the poor fecal sludge and wastewater management; and (5) exploration of existing policies, initiatives, and perspectives for improving this sector. The three interview guides used in each stakeholder group are available in Appendix 1. Institutional stakeholders who participated in the interviews were contacted online using the zoom tool to accommodate their availability while technical and community stakeholders were interviewed face-to-face. Each interview lasted approximately 20 min. The interviews with institutional stakeholders focused mainly on the socio-economic and environmental determinants of emptying practices, as well as the regulatory mechanisms that govern those practices.

### Data analysis

#### Quantitative analysis

The household data was structured in such a way that, for each household, we had specific socioeconomic, housing, and environmental variables (as described in Table 4). Data were analyzed using R studio software version 4.3.0 (Horton and Kleinman 2015). The dependent variable in this research is a binary variable indicating the practice of unhygienic/unsafe emptying by households with a value coded in 1 (unhygienic/unsafe) and 0 (hygienic/safe). This probability value is not optimally expressed in the form of a linear function, Therefore, it was converted to a logit function using the following equation (Eq. (3)) (El Sanharawi and Naudet 2013):

3 $$Ln (P/(1-P))=\text{logit }(P)= {\beta }_{0}+ {\beta }_{1}{X}_{1}+ {\beta }_{2}{X}_{2}+ \cdot \cdot \cdot + {\beta }_{n}{X}_{n}$$

where $P$/ (1 − $P$) is the odds or likelihood ratio representing the ratio between the probability P that the dependent variable is realized and the probability (1 − $P$) that the dependent variable is not; ${\beta }_{0}$ is the intercept (or *y*-intercept); ${X}_{i}$ represents the explanatory variables, and ${\beta }_{i}$ are the coefficients to which these variables are attached.

**Table 4:** Quantitative variables attributed to each household, used to build the logistic regression models

Variable type	Source	Description
Dependent variable	collected during the surveys; *N* = 359 (with valid answers for all variables listed here)	Whether the household resorts to unhygienic/unsafe (binary, 0 = hygienic/safe or 1 = unhygienic/unsafe)
Independent, socio-economic, and environmental variables	Collected during the surveys; *N* = 359 (with valid answers for all variables listed here)	(i) The head of the household achieved secondary or higher education (binary, 0 = no or 1 = yes)
		(ii) The household’s dwelling is classified as “progressive” or “deprived” housing (binary, 0 = no or 1 = yes)
		(iii) The household earns a relatively high monthly income, i.e., twice the Ivorian minimum wage (binary, 0 = no or 1 = yes)
		(iv) The household is accessible by vacuum trucks (binary, 0 = no or 1 = yes)
		(v) Being house owner (binary, 0 = no or 1 = yes)
		(vi) The household lives near a ravine, i.e., within a radius of 100 m (binary, 0 = no or 1 = yes)
	Generated from the buildings’ footprints (secondary data manually updated)	(vii) Distance to closest water body, i.e., river, canal, or lagoon (continuous variable standardized, ranging from 0 to 1 = max distance)
		(viii) Mean area of building footprints within 500 m radius (continuous variable standardized, ranging from 0 to 1)
		(ix) Mean number neighbors within 500 m radius (continuous variable standardized, ranging from 0 to 1)

We assessed associations between the dependent variable and housing, socioeconomic, and environmental characteristics (independent variables) in three steps. First, we conducted bivariate logistic regressions to identify potential explanatory variables. At this step, the significance level was purposively set at a high threshold, i.e., *p*-value < 0.20 (El Sanharawi and Naudet 2013). Thus, in the second step, only variables with a *p*-value < 0.20 were included in a multivariate logistic regression model (LRM). In the latter, we increased the precision level to a significance threshold of 5% (*p*-value ≤ 0.05). In the third step, non-significant variables (*p* > 0.05) were excluded from the model, while those with a better level of significance (*p* ≤ 0.05) were retained in the final, multivariate LRM. In this third step, the level of multicollinearity between the retained variables was assessed through the variance inflation factor (*VIF*). We set a *VIF* threshold value of 3 (Vörösmarty and Dobos 2020) — no variable with a value above this threshold was found.

#### Qualitative analysis

First, during the interviews, the conversations were recorded either directly on the computer (when using Zoom) or with a digital voice recorder (when meeting the informant in person). For each interview the recording was accompanied by notes on the main points discussed. Second, the recordings were systematically transcribed into Microsoft Word and edited to facilitate the analysis by modifying or removing repetitive or unimportant elements. Third, the data were analyzed through coding, categorizing, and matching data, which was then verified and validated checking data (Elo and Kyngäs, 2008). All the transcripts and notes taken during interviews were manually coded based on a preliminary list of codes developed from the research objectives and completed during a careful reading of all the transcripts. Codes can be defined as textual labels in the form of keywords that describe the content of information and are used to capture key thoughts or concepts. The coded segments were grouped and synthesized into themes during content analysis and interpretation.

Content analysis (CA) can be seen as an interpretation of the content of textual data through the systematic classification process of coding and identifying themes or patterns without imposing preconceived categories or theoretical perspectives (Hsieh and Shannon 2005). We used thematic CA (Krippendorff 2018) to understand the institutional framework of FSM, the emptying practices observed in the study area, the operational practices in place, the challenges encountered in the sector and endogenous measures to improve existing conditions. This analysis also allowed assessment of stakeholders’ level of knowledge of existing regulations and the health and environmental risks associated with poor FSM.

## Results

### Characteristics of the population in the study area

- Expression of the variables in the total population
  A total of 559 households were interviewed. Most of them (61.2%) were young people, between the ages of 18 and 35, and more than a half of them were women (61.2%). Secondary education was the highest level attained by most respondents (62.2%), followed by primary education (21.4%) and no formal education (16.4%). Furthermore, in terms of monthly income, 34.9% of respondents reported a relatively high income, i.e., more than double the Ivorian minimum monthly income (a total of 251 USD) (CICG 2022).
  Most of the respondents (72.4%) lived in low-standard housing, i.e., either progressive or deprived housing or 36.1% of the housing compounds organized around courtyards were shared by several households. About 29% of the households were owners and 71% were tenants. The tenants, most of them young, were responsible for hiring services to empty the fecal sludge in the study area. Regarding the environmental characteristics of the households surveyed, 28.1% lived near a gully. In addition, almost a third (29%) of the households located in an area with difficult access by vacuum trucks. This situation was observed not only in deprived neighborhoods but also in progressive and economic housing.
- Expression of the potential explanatory variables of emptying practice
  In the study area, 380 of the 559 households surveyed reported having emptied their pit latrine or septic tank at least once since moving into their dwelling. Of these 380 households, 359 provided valid responses for the study variables. The expression of the variables collected during the surveys in households that have already emptied their pits evolves in practically the same order of magnitude as their expression in the total population, as shown in Table S1 (Appendix 2). This situation clearly shows the validity of the expression of these variables in terms of representativeness to explain the phenomenon under study.
  Concerning the morphological indicators characterizing the respondents’ environment, the spatial analysis revealed that the distance of households to the closest water body varied between 1.71 and 2177 m. Furthermore, the mean area of the buildings around the selected households (within a fixed radius of 500 m) ranged between 61 and 188 m^2^. The selected households had a mean number of neighbors (number of other buildings within a fixed radius of 500 m) ranging between 31 and 273. The environmental characteristics of households in the study area are summarized in Table S2 (in Appendix 2).

### Emptying methods used

**Fig. 2:**
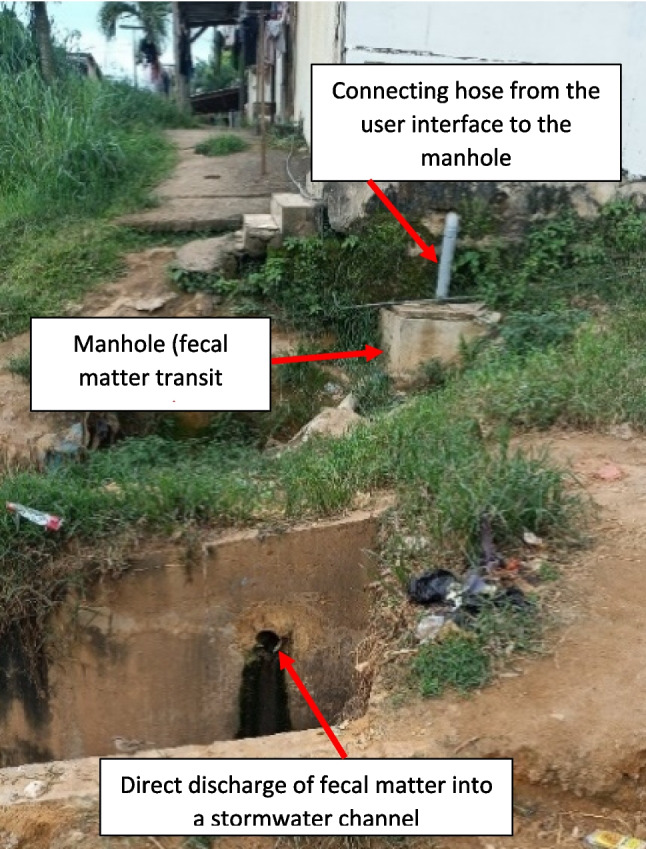
Example of unhygienic/unsafe emptying: emptying of fecal sludge into open drains via illegal connection in study area

**Fig. 3:**
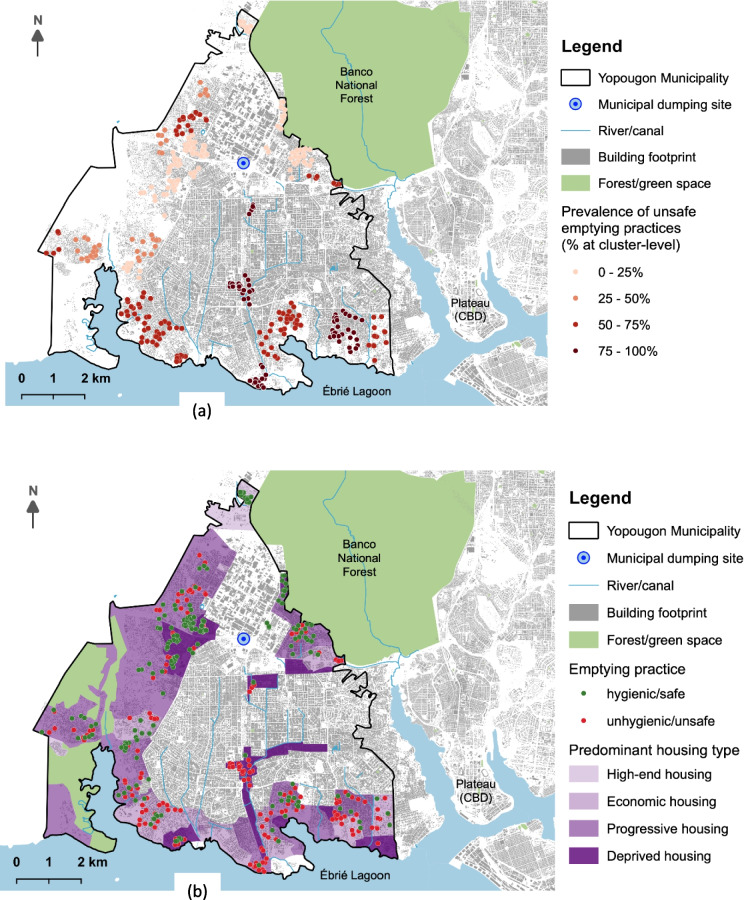
Spatial distribution of hygienic/safe and unhygienic/unsafe fecal sludge emptying practices in the study area: **a** unsafe practice prevalence and **b** emptying practices

In the study area, almost half of all households (47.6%) recurred to unhygienic/unsafe methods to dispose of their fecal sludge. Continuous disposal of sludge through illegal pipe connections to pits or toilets (see Fig. 2) was the most common unhygienic/unsafe practice (38.1%), followed by manual emptying, which was less common (9.5%). As shown in Fig. 3, unhygienic/unsafe emptying practices are observed across the study area with a higher prevalence in the southern part bordered by the Ebrié lagoon (Fig. 3a). These unhygienic/unsafe practices were also observed in all housing types except in one high-end housing subsector located in the west side of the Banco Forest (Fig. 3b).

Hygienic/safe emptying was practiced by 52.3% of households in the study area, mainly relying on mechanical methods using vacuum trucks. Most of these trucks were equipped with a single pump, and only a few had a high-pressure pump allowing a more efficient emptying process. The capacities of the tankers operating in the study area ranged from 6.5 to 15 m^3^. The sludge emptied by these trucks was reported to be transported from households to a dedicated dumping site set up by the authority in charge of sanitation, in accordance with hygiene and safety standards.

### Stakeholders’ perceptions of emptying practices

According to stakeholders from the competent local and national authorities, clear policies have been defined to guide the sanitation sector, reinforced by the creation of a national sanitation office (Office National de l’Assainissement et du Drainage, ONAD) in charge of enforcing the existing regulations. As part of ONAD’s activities, fecal sludge emptying operators have benefited from capacity building programs enabling them to safely manage excreta. At the national level, among others regulation instruments, the Water Code established in 1998 and revised in 2023, regulates sanitation services and institutes penalties for unsafe practices. At the local level, these instruments are reinforced by the Yopougon Municipality Declaration of 2014. However, the local authorities responsible for enforcing the regulations in the field do not have sufficient technical, financial, or human resources to carry out the necessary interventions. According to them, the decentralization of governance and responsibilities, currently in process, has not been effectively accompanied by the decentralization of financial resources, thus limiting their ability to intervene in the sector (information campaigns and enforcement of regulations).

From the perspective of the populations living in the study area, those regulatory instruments are little known, as these populations have not benefited from awareness-raising or information campaigns on the issue. In addition, among the community members interviewed, some argued that the cost of emptying is not well regulated, and the rates charged by emptying companies are beyond the reach of most households, who already struggle to pay the sanitation tax deducted from the water bill. Other community members claimed to be unaware of the health benefits of hygienic emptying, as compared to non-hygienic emptying. They emphasized that with hygienic emptying represented a constant expenditure that was too high, whereas with non-hygienic emptying (illegal toilet/pit connections), they only needed to spend money once, for the installation of the pipes. Those who were aware of the regulations and standards claimed that these instruments failed to provide affordable alternatives to help promoting safe emptying practices.

### Drivers of unhygienic/unsafe emptying practices

The results of the univariate analysis are presented in Table S3 (in Appendix 2). According to this preliminary analysis, the independent variables meeting the threshold significance level (*p* < 0.2) and thus eligible to be included in the multivariate LRM were “household accessible by a vacuum truck” (*p* < 0.001); “household head with high monthly income” (*p* = 0.13); “being house owner” (*p* < 0.001); “household close to gully/gutter” (*p* < 0.001); “distance to closest water body” (*p* < 0.001); “mean area of building footprints” (zonal statistic considering a buffer of 500 m from the household point location, *p* = 0.005), and “'mean number of neighbors”' (zonal statistic considering a buffer of 500 m from the household point location, *p* = 0.009).

The final, multivariate LRM showed that among all these independent variables, “distance to closest water body,” “being house owner,” “household head with high monthly income,” and “household close to gully/gutter” were still significantly associated with the practice of unhygienic/unsafe emptying once accounting for the other variables preselected through the bivariate analysis. Indeed, according to the adjusted odds ratio (*aOR*), the likelihood of practicing unhygienic/unsafe emptying is extremely lower in households located more than 500 m away from the closest water body, than in those that are closer (*aOR* = 0.03; 95% *CI* 0.009–0.12; *p* < 0.001). The same situation is observed regarding housing tenure status. In fact, the likelihood of unhygienic/unsafe emptying is extremely lower in households that own their own houses than in those where they do not (*aOR* = 0.27; 95% CI 0.15–0.47; *p* < 0.001).

This likelihood is however higher in households close to gully or gutter (≤ 100 m), than in those further away (*aOR* = 1.73; 95% *CI* 0.99–3.03; *p* = 0.05). Income level was also statistically linked to the choice of emptying practice, with unhygienic/unsafe emptying being significantly less frequent in wealthy households than in poorer ones (*aOR* = 0.59; 95% *CI* 0.35–0.98; *p* = 0.04). The results of the final multivariate LRM are presented in Table 5.

**Table 5:** Multivariate logistic regression showing association between identified factors and unhygienic/unsafe emptying practice

Characteristic	*OR*	*aOR*	aOR’s 95% *CI*	*p*-value
Household close to gully/gutter	2.42	1.73	0.99–3.03	0.05*
Household accessible by a vacuum truck	0.43	0.62	0.34**–**1.11	0.11
Household head with high monthly income	0.71	0.59	0.35–0.98	0.04*
Being house owner	0.31	0.27	0.15–0.47	< 0.001***
Distance to closest water body	0.03	0.03	0.01–0.13	< .001***
Mean area of building footprints within 500 m	0.06	0.14	0.01**–**1.77	0.13
Mean number of neighbors within 500 m	0.75	0.82	0.09**–**6.96	0.85

*OR* raw odds ratio, *aOR* adjusted odds ratio, *CI* confidence interval

^*^p ≤ 0.05, **p < 0.01, ***p < 0.001

## Discussion

This study highlighted fecal sludge management practices in the municipality of Yopougon and the factors associated with unhygienic/unsafe practices. The latter are essentially characterized by the illegal connection of toilets/pits directly to open drains and manual emptying without transporting the sludge, represent a significant challenge to safely managed sanitation, as recommended by the Sustainable Development Goal (SDG) 6, target 6.2. In fact, in the study area these practices result in the discharge of fecal sludge into the environment without prior treatment. Almost half (47.6%) of the enrolled households recurred to such practices, which have serious repercussions for the environment and public health (Berendes et al. 2020; Mosimane and Kamwi 2020). A study carried out in Bangladesh by Foster et al. (2021) showed that discharging fecal sludge into the environment led to a 68% increase in annual cases of illness linked to exposure to fecal pathogens. Similarly, a recent study carried out in the Abobo commune of Abidjan came to the similar conclusions, showing that the discharge of untreated fecal sludge into the environment is the main contamination route for enteropathogens (P. Z. Z. Coulibaly et al. 2023).

These unhygienic/unsafe emptying practices, most of which (80.1%) were characterized by direct discharge of sludge into open drains via illegal connections, are not specific to the study area and have also been revealed by other authors in other low- and middle-income countries in Africa and Asia (Foster et al. 2021; Mbang et al. 2021; Sultana et al. 2023). The study also revealed that the use of unhygienic/unsafe methods of emptying toilets was not confined to a particular type of dwelling but concerned at least three of the four types of dwelling identified in Yopougon. This implies that the practice observed in low- and middle-income towns is not specific to informal settlements. According to the public authorities in charge of sanitation issues in the study area, regulations exist at both central (Law no. 2023–902, November 23, 2023) and local (deliberation N2014-02/C.Yop/SG, March 20, 2014) levels that do not allow unhygienic/unsafe emptying practices. These regulations seem to be old-fashioned and outdated or not working well due to the occurrence of this practice observed in this current research. For example, at central level, the only article (Art. 51) related to fecal pollution states that “all wastewater discharges into the receiving environment must comply with current standards.” However, there are no national standards or enforcement measures in this area.

In Yopougon, even if unhygienic/unsafe emptying practices are subject to specific fines under current regulations, the lack of legal instruments makes enforcement difficult. This situation, which shows a weakness in terms of regulations, was highlighted by Lerebours et al. (2021). In fact, the results of his study show that even if there are partial regulations on the subject in Abidjan, the texts relating on sanctions remain limited, and there is no data that exists on their implementation. Such an environment is conducive to illegal practices, particularly unhygienic emptying, insofar as it can foster a sense of freedom of action without legal concerns. This observation is almost identical in all Sub-Saharan African countries, where there is a general lack of regulatory frameworks and provisions relating to emptying and fecal sludge transport services (Lerebours et al. 2021).

By considering environmental and socio-economic determinants of emptying practices, the multivariate logistic regression analysis identified three variables were significantly associated with a low likelihood of unhygienic/unsafe practices. These variables were “head of household with high monthly income” (*p* = 0.04); “distance to closest water body” (*p* < 0.0001); and “household own their own house” (*p* < 0.0001). The variable “household close to a gully” (*p* = 0.05) is associated with a high likelihood of unhygienic/unsafe emptying. Among these four variables, “'household own their own house”' and “distance to closest water body” were the highly significant variables (*p* < 0.0001), associated with the practice of emptying. Thus, being the owner of the occupied dwelling and living in a household close to a water body are the most determining factors in terms of emptying practices in Abidjan. Indeed, if the occupant of the household is the owner of the house, the likelihood of unhygienic/unsafe emptying is much lower (*aOR* = 0.27; 95% *CI* 0.15–0.47; *p* < 0.0001). In other words, in rented houses, the likelihood of using unhygienic/unsafe emptying methods, namely illegal connection emptying and manual emptying, is more frequent than in non-leased houses.

In Côte d’Ivoire, according to the current legislation (articles 268 and 431 of Law n2019-576 of June 26, 2019, instituting the Construction and Housing Code), the landlord, is responsible not only for the construction of on-site sanitation facilities but also for emptying them when they are full. The landlord’s commitment to this responsibility affects his savings, since he must spend money on hygienic/safe (mechanical) emptying of the pits, the regularity of which depends on how often the facilities are filled. This indicates that the owner is a central element in the system and, in this sense, influences emptying practices in the study area.

As for the association between high income and safe practices, in the study area, the average cost per rotation for mechanical emptying is between 42 and 51 USD, which is relatively high, as the key informants pointed out during the interviews. In addition, the required frequency of emptying in the area is quite high (3 times a year on average), which increases the cost related to emptying fecal sludge. Previous studies have come to similar conclusions regarding the high cost of mechanical emptying in Sub-Saharan Africa. In Dakar, Senegal, households’ willingness to pay for mechanical emptying varies in the same direction as their wealth index (Gning et al. 2017; Scott et al. 2013). In the city of Bafoussam in Cameroon, where emptying is carried out by private operators like in Abidjan, the cost of emptying varies from 42 to over 93 USD (Defo et al. 2015). These costs are even higher than in Côte d’Ivoire and Senegal, thus constituting significant barriers to safe practices. In Maputo, Mozambique, the high cost of hygienic/safe mechanical emptying (25 to 50 USD) compared with unhygienic/unsafe manual emptying (8 to 17 USD) was also highlighted (Capone et al. 2020). In short, the relatively high cost of mechanical methods seems to be a common barrier to safe practices of fecal sludge management in Sub-Saharan African.

Hence, economic factors may explain why applicants for pit emptying services (PES) turn to less safe alternatives. Indeed, as landlords seeking to make a profit, owners would be interested in advantageous solutions that reduce their costs and allow them to maximize their profits. Under these conditions, manual emptying and, especially illegal connections are attractive alternatives for landlords. In the case of illegal connections (the most common method of unhygienic/unsafe emptying), only the installation cost is borne by the landlord, who does not have to spend any more money for the rest of the life cycle of the installation. Similarly, the low cost of manual, unhygienic/unsafe emptying (44 to 51 USD), compared with the cost of mechanical, hygienic/safe emptying (126 to 153 USD), observed in the study area, could make this practice a preferred choice for applicants’ PES. Previous studies have shown that manual emptying is preferred to mechanical emptying due to its affordability. This is the case in Dar Es Salaam in Tanzania where households favor the option of manual emptying in favor of mechanical emptying due to its exorbitant cost (Van Dijk et al. 2014).

The other highly significant variable associated with the practice of emptying, namely “distance to closest water body,” is expressed in the same way as the variable “'household own their own house.” Again, the cost of emptying is central and influences the choice between hygienic/safe and unhygienic/unsafe practices. In the study area, the likelihood of practicing unhygienic/unsafe emptying increases with distance to the nearest water source increases (*aOR* = 0.03; *CI* 0.009–0.12; *p* < 0.001), with a critical threshold around 500 m. It was observed that households living close to a canal, river, or lagoon tend to use these bodies of water or canals as receptacles for their fecal sludge. For these households, this type of unhygienic/unsafe practice is an easy and accessible method that, unlike others, does not require periodic maintenance costs and is therefore less stressful. With this method, the effluent and sludge are discharged directly and continuously into the receiving environment, so that the pits/tanks almost never fill up and the toilets always remain operational. This could explain the association between the variable “household close to a gully” (*p* = 0.05) and a higher likelihood of unhygienic/unsafe emptying.

The unhygienic/unsafe practices prevailing in the study area seem to be linked to the search for affordable solutions but that neglect aspects related to environmental hazards. Our findings strongly suggest the need to raise public awareness of the importance of adopting good sanitation practices in general and fecal sludge management in particular. They also show that awareness-raising campaigns, which have so far focused on vulnerable and low-income neighborhoods, need to target all social strata. In fact, the type of housing (“deprived” or “progressive”) had no significant influence on the type of sanitation practice (*p*-value > 0.2, detected in the univariate analysis). In addition, awareness-raising interventions should give particular emphasis to residents of areas near water bodies.

Our findings also suggest there is a need to subsidize the costs of mechanical (hygienic/safe) emptying to facilitate its adoption by applicants’ PES. Indeed, according to the WHO, well-designed, transparent, and targeted subsidies would make it possible to effectively combat financial constraints on the practice of hygienic emptying. On the other hand, ill-conceived subsidies risk serving the rich rather than the poor (World Health Organization 2020). Moreover, the implementation of subsidies would first have to comply with a number of provisions, in particular the adoption of a single emptying tariff, which is not the case in our context. A preliminary study of the determinants governing the mechanical (hygienic/safe) emptying tariff is necessary for its approval. While it is true that there is no shortage of tanker trucks in the study area (over 30 trucks with capacities ranging from 6.5 to 15 m^3^), other information such as the average distance travelled to reach the dumping site, and operating costs need to better understand.

While awareness-raising and subsidies appear to be key to improving the emptying situation, the sustainability of interventions lies in the application of effective regulatory measures. A recent study by Ahmed et al. (2022) found that national policy and funding are recommended in WASH interventions. Indeed, regulatory mechanisms are necessary to influence the population’s incentive to adopt safer behaviors. The importance of sanitation regulations has been understood by most decision-makers in Sub-Saharan Africa. However, there are difficulties in enforcing these regulations (Lerebours et al. 2021). In Côte d’Ivoire, the national level, the Water Code also contains provisions in this area (Articles 48 and 49), prohibiting the discharge of wastewater into the receiving environment without prior treatment. A local initiative has also been taken in the study area by the municipal council to regulate sludge emptying practices. For this purpose, a fee of 59 USD has been imposed on all offenders. However, the prevalence of unhygienic/unsafe practices in the area, despite these regulations confirms the difficulties in implementing the various regulations. These limitations include a lack of sufficient communication on these regulations and poor implementation on the ground. Elsewhere in Africa, difficulties in implementing and enforcing sanitation laws and regulations in the sanitation sector have also been observed. In Tanzania, for example, a study highlighted the fact that regulations on good emptying practices are not producing the expected results in terms of improvements, due to their obsolescence (Seleman et al. 2020). Strengthening regulations for good sanitation practices can count on an increasingly supportive policy in Africa.

Until recently, there were no specific sanitation programs but rather integrated water and sanitation programs. The inadequate integration of sanitation into these programs has resulted in little attention being paid to the sector. In fact, better results could have been achieved if these programs had been effectively supported and implemented (Narayan et al. 2021).

Since the eThekwini Declaration and Africa San Action Plans adopted at the Second African Conference on Sanitation and Hygiene in Durban (Union 2008), the African governments committed to invest 0.5% of their GDP in sanitation. This commitment toward increasing access to safe sanitation services has been reinforced by the SDGs, particularly target SDG 6.2. As a result of this commitment, and with the support of technical and financial partners, several sanitation programs have been implemented in Sub-Saharan Africa, with a strong emphasis on fecal sludge management. This is illustrated by creation of ministries specifically dedicated to sanitation in several countries such as Côte d’Ivoire. Although much remains to be done both in Côte d’Ivoire and in other Sub-Saharan African countries, as most of these countries have failed to meet this commitment (Weststrate 2023), it is a promising paradigm shift to ensure concrete and specific improvements in sanitation sector in Africa.

## Study limitations

We conducted a cross-sectional analysis enabling to verify correlations between unhygienic/unsafe emptying practices observed in the study area at a specific time and the environmental and socio-economic characteristics of the households surveyed. This study design is limited to demonstrating the existence of associations between variables, without establishing cause-and-effect relationships. To address this limitation, we conducted interviews with key stakeholders to enhance our understanding of the relations found between the outcome of interest and the explanatory variables. In addition, further studies on the determinants of the price of mechanical (hygienic) emptying in the study area are needed to identify sustainable hygienic emptying practices and subsidy mechanisms to be put in place.

## Conclusion

Unhygienic/unsafe emptying of fecal sludge hinders the achievement of safely managed sanitation, as envisaged by the SDG target 6.2. Nonetheless, this study revealed that almost half (47.6%) of the enrolled households recurred to such practices, mostly through illegal, piped connections dumping wastewater directly in the nature. Despite the existence of a law on the water code and a municipal declaration, these hazardous practices continue to develop. Improving the health situation requires a change in behavior at all levels. Addressing this sanitation challenge requires effective communication, adapted regulations and the means to enforce them. Notably, it is necessary to intensify public awareness campaigns and implement a subsidy strategy, especially for poorer households, lowering the costs of mechanical emptying, thus making safe fecal sludge management accessible to all.

Our findings provide empirical evidence on the determinants of safe (hygienic) fecal sludge management in Abidjan. There, unhygienic/unsafe practices were not associated with a particular type of housing, although in the wealthy households, this practice was significantly less frequent than in poorer ones. Having a household head with a high monthly income, living far (over 500 m) from the closest water body, and being a house owner were the main variables associated with safe emptying practices. This likelihood increased when the household was close (≤ 100 m) to gully/gutter. Therefore, the socioeconomic context and some environmental factors (distance to water body, gully, or gutter) were identified as key determinants of fecal sludge management practices.

## Supplementary Information

Below is the link to the electronic supplementary material.

Supplementary file1 (DOCX 32 KB)

Supplementary file2 (DOCX 20 KB)

Supplementary file3 (DOCX 18 KB)

Supplementary file4 (DOCX 20 KB)

## Data Availability

The datasets used and/or analyzed during the current study are available from the corresponding author on reasonable request.
