# Supplementary material for: Environmental and socio-economic determinants of fecal sludge emptying in Sub-Saharan Africa: A cross-sectional mixed-methods study in Abidjan, Côte d’Ivoire
Source: Environ Sci Pollut Res Int. 2024 Dec 5;31(58):66497–511. doi: 10.1007/s11356-024-35631-6 (PMC11659383; doi:10.1007/s11356-024-35631-6)
Supplement: Supplementary file 1 — Supplementary file1 (DOCX 32 KB) [file 11356_2024_35631_MOESM1_ESM.docx]

**APPENDIX 1. INTERVIEW GUIDES**

**Article title:** Environmental and socio-economic determinants of fecal sludge emptying in sub-Saharan Africa: a cross-sectional mixed-methods study in Abidjan, Côte d’Ivoire.

**Journal name:** Environmental Sciences and Pollution Research

**Author names and affiliation:**

**Lou Tinan Ange-Laetitia Tra*^1,2^, Kouassi Dongo^1,2^, Vitor Pessoa Colombo^3^,** **Shirish Singh^4^, [Jérôme Chenal](https://www.eawag.ch/en/about-us/portrait/organisation/staff/profile/linda-strande/show)^[3,5](https://www.eawag.ch/en/about-us/portrait/organisation/staff/profile/linda-strande/show)^**

***^1^*** *Département Recherches et Développement (DRD), Centre Suisse de Recherches Scientifiques en Côte d’Ivoire (CSRS), 01 BP 1303 Abidjan 01, Côte d’Ivoire.*

***^2^*** *Laboratoire des Sciences du Sol, de l’Eau et des Géo matériaux (LSSEG), Ecole Doctorale STAD, Université Félix Houphouët-Boigny, 01 BP V34 Abidjan 01, Côte d’Ivoire.*

***^3^*** *Communauté d’Etudes pour l’Aménagement du Territoire, Ecole Polytechnique Fédérale de Lausanne (EPFL),* Bâtiment BP – Station 16 CH-1015 Lausanne*,* Suisse.

***^4^****IHE Delft Institute for Water Education, PO Box 3015, 2601 DA Delft, The Netherlands.*

***^5^****Center of Urban Systems (CUS),* *University Mohammed VI Polytechnic (UM6P), Benguerir 43150, Morocco.*

*Corresponding author

**Lou Tinan Ange-Laetitia TRA**

**E-mail address of the corresponding author:** [tralou.angel@gmail.com/](mailto:tralou.angel@gmail.com/) [ange.tralou@csrs.ci](mailto:ange.tralou@csrs.ci)

**INTERVIEW GUIDE FOR PUBLIC SERVICE PLAYERS**

- *Start of interview:* H *Interview date:*

**Study introduction:**

Dear Madam/Mr.

My name is Ange-Laetitia TRA Lou. I'm a PhD student in sanitation at the Université Félix HOUPHOUET-BOIGNY in Cocody. I'm working on issues related to the sustainable management of fecal sludge (MFS) in the municipality of Yopougon. The aim of the study we are conducting is to see how access to septic tank emptying services in the city of Abidjan can be improved using digital tools for monitoring and decision-making. To do this, we need to talk to you, the key players, to better understand the existing situation in terms of septic tank emptying practices in Abidjan and Yopougon in particular.

Thank you once again for your precious time for this interview, which will only last about twenty minutes.

| **LIBELS AND INSTRUCTIONS** | **CATEGORY/MODALITY CODE** |
| --- | --- |
| **BACKGROUND INFORMATION** | |
| Full Name (interviewee) |  |
| Structure |  |
| Position (interviewee) |  |
| Years of experience in the sanitation sector |  |
| Gender (sex) |  |
| **INSTITUTIONAL AND REGULATORY FRAMEWORK** | |
| General presentation of the structure (mission and organization chart) |  |
| Presentation of the department or sub-department to which the interviewee belongs |  |
| What can you tell us about the current state of fecal sludge management in Abidjan and Yopougon in particular? The players involved and their roles ? |  |
| What can you tell us about the regulations and codes that exist for sanitation and fecal sludge management in particular? |  |
| Coverage rate of on-site sanitation in Abidjan and Yopougon in particular (% not connected to the wastewater network). |  |
| **PRACTICES FOR EMPTYING PITS AND DISPOSING OF EXCREMENT AND WASTEWATER** | |
| The organization of pit-emptying activities in Abidjan and Yopougon in particular. |  |
| Pits emptying practices in Abidjan and materials/equipment used. |  |
| Relations with mechanical emptying companies and difficulties encountered. |  |
| Relations with manual emptiers and difficulties encountered. |  |
| Other forms of emptying or disposal of wastewater and excrement practiced by households (operating mode). |  |
| In your opinion, what are the health and environmental risks associated with poor wastewater and excreta management? |  |
| Reasons for unauthorized emptying. |  |
| Measures taken by the State to try and deal with these practices. |  |
| Are there active programs for: (i) promotion of safe on-site sanitation?  (ii) behavior change and community involvement (awareness-raising)? |  |
| Challenges linked to the emptying activity in Abidjan and Yopougon in particular. |  |
| What do you consider to be the greatest difficulties you encounter in managing fecal sludge? |  |
| In your opinion, how can the management of fecal sludge in Abidjan be improved by considering every link in the value chain? |  |

- *End of interview :* H

**N.B: Always thank the participant at the end and take a photo with his/her consent.**

**INTERVIEW GUIDE FOR EMPTYING SERVICE PROVIDERS**

- *Start of interview:* H *Interview date:*

**Study introduction:**

Dear Madam/Mr.

My name is Ange-Laetitia TRA Lou. I'm a PhD student in sanitation at the Université Félix HOUPHOUET-BOIGNY in Cocody. I'm working on issues related to the sustainable management of fecal sludge (MFS) in the municipality of Yopougon. The aim of the study we are conducting is to see how access to septic tank emptying services in the city of Abidjan can be improved using digital tools for monitoring and decision-making. To do this, we need to talk to you, the key players, to better understand the existing situation in terms of septic tank emptying practices in Abidjan and Yopougon in particular.

Thank you once again for your precious time for this interview, which will only last about twenty minutes.

| **LIBELS AND INSTRUCTIONS** | **CATEGORY/MODALITY CODE** |
| --- | --- |
| **BACKGROUND INFORMATION** | |
| Full Name (interviewee) |  |
| Emptying company name |  |
| Position within the company (truck owners or  truck drivers) |  |
| Professional experience in the pit emptying sector (number of years) |  |
| Genre (sexe) |  |
| **PRACTICES FOR EMPTYING PITS AND DISPOSING OF EXCREMENT AND WASTEWATER** | |
| Who do you think is involved in sanitation and pit-emptying issues in Abidjan in general and Yopougon in particular? |  |
| What's the role of each? |  |
| As pit emptiers, what do you actually do? |  |
| Do you work with the State (at central level as well as with the municipality)? |  |
| How are your relationships going? |  |
| What do you gain from working with the government? |  |
| Have you (your employees) already benefited from training programs to strengthen your capacity as a service provider? |  |
| If so, what training courses were involved? |  |
| Which of these were organized by the state or the municipality? |  |
| Do you think they were necessary for you? Why or why not ? |  |
| What means do you think people use to empty their pits? |  |
| In your opinion, are any of these emptying techniques not authorized by the State? |  |
| If so, which ones ? |  |
| What do you know about the method of emptying pits through pipe connections? |  |
| Do you know whether this type of practice is accepted by the state? |  |
| How do you find this type of emptying (good or bad)? Why or why not?  In your opinion, can this kind of practice make you sick? Why or why not? What kinds of illnesses can it create? |  |
| Do you think it’s only people living in deprived neighborhoods who indulge in this practice? |  |
| In your opinion, what motivates people not to use your method (emptying by truck) to the detriment of others (manual, pipe connections)? |  |
| Do you know if there are any laws in Côte d'Ivoire that punish these dumping practices? At municipal level? |  |
| Do you know if there are any laws in Côte d'Ivoire that punish these dumping practices? At municipal level? |  |
| If so, what do you know about these laws? |  |
| How did you find out about them? |  |
| In your opinion, what diseases are linked to poor wastewater and excreta management? |  |
| What difficulties (problems) do you encounter in your business? |  |
| What do you think needs to be done to improve the pit-emptying sector in Abidjan? |  |

- *End of interview :* H

**N.B: Always thank the participant at the end and take a photo with his/her consent.**

**INTERVIEW GUIDE FOR COMMUNITY LEADERS**

- *Start of interview**:* H *Interview date:*

**Study introduction:**

Dear Madam/Mr.

My name is Ange-Laetitia TRA Lou. I'm a PhD student in sanitation at the Université Félix HOUPHOUET-BOIGNY in Cocody. I'm working on issues related to the sustainable management of fecal sludge (MFS) in the municipality of Yopougon. The aim of the study we are conducting is to see how access to septic tank emptying services in the city of Abidjan can be improved using digital tools for monitoring and decision-making. To do this, we need to talk to you, the key players, to better understand the existing situation in terms of septic tank emptying practices in Abidjan and Yopougon in particular.

Thank you once again for your precious time for this interview, which will only last about twenty minutes.

| **LIBELS AND INSTRUCTIONS** | **CATEGORY/MODALITY CODE** |
| --- | --- |
| **BACKGROUND INFORMATION** | |
| Full Name (interviewee) |  |
| Housing type concerned |  |
| Name of community association or syndicate |  |
| Position within the community structure (interviewee) |  |
| General presentation of the structure (mission and organization chart) |  |
| Time spent in the neighborhood (number of years) |  |
| Gender (sex) |  |
| **PRACTICES FOR EMPTYING PITS AND DISPOSING OF EXCREMENT AND WASTEWATER** | |
| Who do you think is involved in wastewater and excreta issues in Abidjan in general and in your neighborhood in particular? |  |
| What's the role of each? |  |
| What do you do when septic tanks are full? Who takes care of this? How do they do it? |  |
| In your opinion, is there a particular type of emptying that is accepted by the state? |  |
| Is the discharge of sludge into the environment via connections part of this type of state-approved emptying? |  |
| How do you find this type of emptying (good or bad)? Why or why not ? |  |
| In your opinion, can this kind of practice make you sick? Why or why not? What kinds of illnesses can it create? |  |
| Do you think it’s only people living in deprived neighborhoods who indulge in this practice? |  |
| Is such a practice common in your community? |  |
| What do you think motivates people to do it? |  |
| Do you know if there are any laws in Côte d'Ivoire that punish these dumping practices? At municipal level? |  |
| If so, what do you know about these laws? |  |
| How did you find out about them? |  |
| Have you ever been made aware of sanitation issues in your community? |  |
| If so, what exactly was involved? |  |
| Have you addressed issues related to pit-emptying practices and the associated risks? |  |
| How do you think the sanitation situation could be improved in your neighborhood? |  |

- *End of interview :* H

**N.B: Always thank the participant at the end and take a photo with his/her consent.**
