# Supplementary material for: Environmental and socio-economic determinants of fecal sludge emptying in Sub-Saharan Africa: A cross-sectional mixed-methods study in Abidjan, Côte d’Ivoire
Source: Environ Sci Pollut Res Int. 2024 Dec 5;31(58):66497–511. doi: 10.1007/s11356-024-35631-6 (PMC11659383; doi:10.1007/s11356-024-35631-6)
Supplement: Supplementary file 2 — Supplementary file2 (DOCX 20 KB) [file 11356_2024_35631_MOESM2_ESM.docx]

**APPENDIX 2. TABLE S1**

**Article title:** Environmental and socio-economic determinants of fecal sludge emptying in sub-Saharan Africa: a cross-sectional mixed-methods study in Abidjan, Côte d’Ivoire.

**Journal name:** Environmental Sciences and Pollution Research

**Author names and affiliation:**

**Lou Tinan Ange-Laetitia Tra*^1,2^, Kouassi Dongo^1,2^, Vitor Pessoa Colombo^3^,** **Shirish Singh^4^, [Jérôme Chenal](https://www.eawag.ch/en/about-us/portrait/organisation/staff/profile/linda-strande/show)^[3,5](https://www.eawag.ch/en/about-us/portrait/organisation/staff/profile/linda-strande/show)^**

***^1^*** *Département Recherches et Développement (DRD), Centre Suisse de Recherches Scientifiques en Côte d’Ivoire (CSRS), 01 BP 1303 Abidjan 01, Côte d’Ivoire.*

***^2^*** *Laboratoire des Sciences du Sol, de l’Eau et des Géo matériaux (LSSEG), Ecole Doctorale STAD, Université Félix Houphouët-Boigny, 01 BP V34 Abidjan 01, Côte d’Ivoire.*

***^3^*** *Communauté d’Etudes pour l’Aménagement du Territoire, Ecole Polytechnique Fédérale de Lausanne (EPFL),* Bâtiment BP – Station 16 CH-1015 Lausanne*,* Suisse.

***^4^****IHE Delft Institute for Water Education, PO Box 3015, 2601 DA Delft, The Netherlands.*

***^5^****Center of Urban Systems (CUS),* *University Mohammed VI Polytechnic (UM6P), Benguerir 43150, Morocco.*

*Corresponding author

**Lou Tinan Ange-Laetitia TRA**

**E-mail address of the corresponding author:** [tralou.angel@gmail.com/](mailto:tralou.angel@gmail.com/) [ange.tralou@csrs.ci](mailto:ange.tralou@csrs.ci)

**Table S1.** Socio-economic characteristics of the respondents

| **Variable** | **Item** | **Frequency of variables in the total population (%)** | **Frequency of potential explicative variables (%)** |
| --- | --- | --- | --- |
| **Sex of respondent** | Male | 38.8 | - |
|  | Female | 61.2 |  |
| **Age of respondent** | 18-35 years | 56.5 | - |
|  | 36-49 years | 27.9 |  |
|  | >50 years | 15.6 |  |
| **Education level** | No formal education | 16.4 | 15.6 |
|  | Primary school | 21.4 | 22.3 |
|  | Secondary and higher | 62.2 | 62.1 |
| **Habitat type** | Deprived housing | 16.1 | 21.5 |
|  | Progressive class housing | 56.3 | 49 |
|  | Economic housing | 21.3 | 25.9 |
|  | High-end housing | 6.3 | 3.6 |
| **Building type** | Individual courtyard | 63.9 | - |
|  | Shared courtyard | 36.1 |  |
| **Being house owner** | Tenants | 70.7 | 72.1 |
|  | Owners | 29.3 | 27.9 |
| **Household head monthly income** | <150.000 FCFA (<251 USD) | 65.1 | 66.3 |
|  | >150.000 FCFA (>251 USD) | 34.9 | 33.7 |
| **Household close to gully/gutter** | No | 71.9 | 71.3 |
|  | Yes | 28.1 | 28.7 |
| **Household accessible by vacuum truck** | No | 29 | 27.6 |
|  | Yes | 71 | 72.4 |
