# Supplementary material for: Environmental and socio-economic determinants of fecal sludge emptying in Sub-Saharan Africa: A cross-sectional mixed-methods study in Abidjan, Côte d’Ivoire
Source: Environ Sci Pollut Res Int. 2024 Dec 5;31(58):66497–511. doi: 10.1007/s11356-024-35631-6 (PMC11659383; doi:10.1007/s11356-024-35631-6)
Supplement: Supplementary file 3 — Supplementary file3 (DOCX 18 KB) [file 11356_2024_35631_MOESM3_ESM.docx]

**APPENDIX 2. TABLE S2**

**Article title:** Environmental and socio-economic determinants of fecal sludge emptying in sub-Saharan Africa: a cross-sectional mixed-methods study in Abidjan, Côte d’Ivoire.

**Journal name:** Environmental Sciences and Pollution Research

**Author names and affiliation:**

**Lou Tinan Ange-Laetitia Tra*^1,2^, Kouassi Dongo^1,2^, Vitor Pessoa Colombo^3^,** **Shirish Singh^4^, [Jérôme Chenal](https://www.eawag.ch/en/about-us/portrait/organisation/staff/profile/linda-strande/show)^[3,5](https://www.eawag.ch/en/about-us/portrait/organisation/staff/profile/linda-strande/show)^**

***^1^*** *Département Recherches et Développement (DRD), Centre Suisse de Recherches Scientifiques en Côte d’Ivoire (CSRS), 01 BP 1303 Abidjan 01, Côte d’Ivoire.*

***^2^*** *Laboratoire des Sciences du Sol, de l’Eau et des Géo matériaux (LSSEG), Ecole Doctorale STAD, Université Félix Houphouët-Boigny, 01 BP V34 Abidjan 01, Côte d’Ivoire.*

***^3^*** *Communauté d’Etudes pour l’Aménagement du Territoire, Ecole Polytechnique Fédérale de Lausanne (EPFL),* Bâtiment BP – Station 16 CH-1015 Lausanne*,* Suisse.

***^4^****IHE Delft Institute for Water Education, PO Box 3015, 2601 DA Delft, The Netherlands.*

***^5^****Center of Urban Systems (CUS),* *University Mohammed VI Polytechnic (UM6P), Benguerir 43150, Morocco.*

*Corresponding author

**Lou Tinan Ange-Laetitia TRA**

**E-mail address of the corresponding author:** [tralou.angel@gmail.com/](mailto:tralou.angel@gmail.com/) [ange.tralou@csrs.ci](mailto:ange.tralou@csrs.ci)

**Table S2.**  Environmental (morphological) indicators characteristic of the respondents’-built environment (standardized continuous variables)

| **Morphological indicators** | **Minimum value** | | **Maximum value** | | **Mean value** |
| --- | --- | --- | --- | --- | --- |
|  | Standardized | Real | Standardized | Real |  |
| **Distance to closest water body** | 0.0007 | 1.71 m | 1 | 2177 m | 596 m |
| **Mean area of building footprints (within 500m)** | 0.32 | 61 | 1 | 188 | 108 |
| **Mean number neighbors (within 500 m)** | 0.11 | 31 | 1 | 273 | 112 |
