# Supplementary material for: Environmental and socio-economic determinants of fecal sludge emptying in Sub-Saharan Africa: A cross-sectional mixed-methods study in Abidjan, Côte d’Ivoire
Source: Environ Sci Pollut Res Int. 2024 Dec 5;31(58):66497–511. doi: 10.1007/s11356-024-35631-6 (PMC11659383; doi:10.1007/s11356-024-35631-6)
Supplement: Supplementary file 4 — Supplementary file4 (DOCX 20 KB) [file 11356_2024_35631_MOESM4_ESM.docx]

**APPENDIX 2. TABLE S3**

**Article title:** Environmental and socio-economic determinants of fecal sludge emptying in sub-Saharan Africa: a cross-sectional mixed-methods study in Abidjan, Côte d’Ivoire.

**Journal name:** Environmental Sciences and Pollution Research

**Author names and affiliation:**

**Lou Tinan Ange-Laetitia Tra*^1,2^, Kouassi Dongo^1,2^, Vitor Pessoa Colombo^3^,** **Shirish Singh^4^, [Jérôme Chenal](https://www.eawag.ch/en/about-us/portrait/organisation/staff/profile/linda-strande/show)^[3,5](https://www.eawag.ch/en/about-us/portrait/organisation/staff/profile/linda-strande/show)^**

***^1^*** *Département Recherches et Développement (DRD), Centre Suisse de Recherches Scientifiques en Côte d’Ivoire (CSRS), 01 BP 1303 Abidjan 01, Côte d’Ivoire.*

***^2^*** *Laboratoire des Sciences du Sol, de l’Eau et des Géo matériaux (LSSEG), Ecole Doctorale STAD, Université Félix Houphouët-Boigny, 01 BP V34 Abidjan 01, Côte d’Ivoire.*

***^3^*** *Communauté d’Etudes pour l’Aménagement du Territoire, Ecole Polytechnique Fédérale de Lausanne (EPFL),* Bâtiment BP – Station 16 CH-1015 Lausanne*,* Suisse.

***^4^****IHE Delft Institute for Water Education, PO Box 3015, 2601 DA Delft, The Netherlands.*

***^5^****Center of Urban Systems (CUS),* *University Mohammed VI Polytechnic (UM6P), Benguerir 43150, Morocco.*

*Corresponding author

**Lou Tinan Ange-Laetitia TRA**

**E-mail address of the corresponding author:** [tralou.angel@gmail.com/](mailto:tralou.angel@gmail.com/) [ange.tralou@csrs.ci](mailto:ange.tralou@csrs.ci)

**Table S3.** Results of bivariate logistic regressions to identify variables potentially associated with unhygienic/unsafe emptying (step 1).

| **Variable** | **OR** | | **95%CI** | **p-value** |
| --- | --- | --- | --- | --- |
| **Secondary/higher education attained** | |  |  |  |
| No (intercept) | | 1 |  |  |
| Yes | | 0.9 | 0.58 – 1.37 | 0.62 |
| **Household lives in low-standard housing** | |  |  |  |
| No (intercept) | | 1 |  |  |
| Yes | | 0.97 | 0.61 – 1.53 | 0.9 |
| **Household head with high monthly income** | |  |  |  |
| No (intercept) | | 1 |  |  |
| Yes | | 0.71 | 0.45 – 1.11 | 0.13* |
| **Being house owner** | |  |  |  |
| No (intercept) | | 1 |  |  |
| Yes | | 0.31 | 0.18 – 0.51 | <0.001*** |
| **Household accessible by a vacuum truck** | |  |  |  |
| No (intercept) | | 1 |  |  |
| Yes | | 0.43 | 0.26 – 0.69 | <0.001*** |
| **Household close to gully/gutter** | |  |  |  |
| No (intercept) | | 1 |  |  |
| Yes | | 2.42 | 1.51 – 3.90 | <0.001*** |
| **Distance to closest water body** | |  |  |  |
| No (intercept) | | 1 |  |  |
| Yes | | 0.03 | 0.009 – 0.10 | <0.001*** |
| **Mean area of building footprints (within 500 m)** | |  |  |  |
| No (intercept) | | 1 |  |  |
| Yes | | 0.06 | 0.008 – 0.42 | 0.005** |
| **Mean number of neighbors (within 500 m)** | |  |  |  |
| No (intercept) | | 1 |  |  |
| Yes | | 7.75 | 1.67 – 37.89 | 0.009** |
| OR= raw Odds Ratio; CI= Confidence Interval; *p<0.2, **p<0.01, ***p<0.001 | | | | |
